# The Bicycle in Surgery

**Published:** 1898-05-14

**Authors:** 


### THE BICYCLE IN SURGERY.

Several modifications of the bicycle were described a short time ago at one of the Berlin Medical Societies, which have recently been introduced with the object of making the machine serviceable in the treatment of morbid conditions, more especially of the joints. Cases of partial ankylosis in the hip, the knee, and more particularly in the ankle, not infrequently occur in which the frequent repetition of certain strictly limited movements is distinctly beneficial. The great point is that the movement be limited in range so as never to over-pass the point at which pain and spasm are produced, and it is here that the accurate adjustment which is possible in the mechanical arrangement of the cycle becomes of service, for it is easy, by arranging the gear and the length of stroke of the pedals to suit the power of the affected side, to obtain just the range of movement which is required for the particular case. For those who are skilled riders these adjustments can be made on the bicycle, but where there is any question about the capacity of the rider, or in those cases in which the patient has not been accustomed to the use of the machine, a well-arranged tricycle should be employed for the purpose. There are even cases in which at first the assistance of an attendant may be required, the movement of the joints being almost purely passive. It may generally be stated that anyone who is able to run the machine with one foot will, by properly adjusting the pedal on the ailing side, be able to bring the method into play if his malady should be one suitable for these exercises. The cases for which this form of mechanical movement seems more especially useful are those of stiffness following sprain of ligaments or septic inflammation of joints, or in which contraction of muscles has taken place in consequence of prolonged confinement in splints.

